# On the effects of program slicing for vulnerability detection during code inspection

**DOI:** 10.1007/s10664-025-10636-y

**Published:** 2025-04-05

**Authors:** Aurora Papotti, Katja Tuma, Fabio Massacci

**Affiliations:** 1 Foundational Security, Afdeling Informatica, Vrije Universiteit, Amsterdam, NL the Netherlands; 2 Dipartimento di Ingegneria e Scienza dell’Informazione, Università di Trento, Trento, IT Italy

**Keywords:** program slicing, vulnerability, code review, program comprehension, controlled experiment

## Abstract

Slicing is a fault localization technique that has been proposed to support debugging and program comprehension. Yet, its empirical effectiveness during code inspection by humans has received limited attention. The goal of our study is two-fold. First, we aim to define what it means for a code reviewer to identify the vulnerable lines correctly. Second, we investigate whether reducing the number of to-be-inspected lines by method-level slicing supports code reviewers in detecting security vulnerabilities. We propose a novel approach based on the notion of a $\delta$-neighborhood (intuitively based on the idea of the context size of the command git  diff) to define correctly identified lines. Then, we conducted a multi-year controlled experiment (2017-2023) in which MSc students attending security courses ($n=236$) were tasked with identifying vulnerable lines in original or sliced Java files from Apache Tomcat. We provide perfect seed lines for a slicing algorithm to control for confounding factors. Each treatment differs in the pair (Vulnerability, Original/Sliced) with a balanced design with vulnerabilities from the OWASP Top 10 2017: A1 (Injection), A5 (Broken Access Control), A6 (Security Misconfiguration), and A7 (Cross-Site Scripting). To generate smaller slices for human consumption, we used a variant of intra-procedural thin slicing. We report the results for $\delta = 0$ which corresponds to exactly matching the vulnerable ground truth lines, and $\delta = 3$ which represents the scenario of identifying the vulnerable area. For both cases, we found that slicing helps in ‘finding something’ (the participant has found at least some vulnerable lines) as opposed to ‘finding nothing’. For the case of $\delta = 0$ analyzing a slice and analyzing the original file are statistically equivalent from the perspective of lines found by those who found something. With $\delta = 3$ slicing helps to find more vulnerabilities compared to analyzing an original file, as we would normally expect. Given the type of population, additional experiments are necessary to be generalized to experienced developers.

## Introduction

Developers are often struggling to deliver secure products, either due to a lack of resources, difficulty in prioritizing security, or knowledge gaps (Weir et al. 2023). Therefore, it is interesting to study to what extent existing techniques can be used for developers without a strong security background to be able to quickly detect the most common vulnerabilities. As an example, source code is inspected for security as part of code review activity, and using static analysis tools (e.g., Linters and heuristic checkers) is a common industry practice to support this manual task. In this paper, we follow Hirsch and Hofer (2021) and focus on the fault localization phase (as opposed to fault remediation phase).

Among the different techniques used for fault localization (Wong et al. 2016) slicing has obtained some positive results in the identification of failures in programs. Weiser proposed the notion of slicing in 1979 (Weiser 1981): extracting program parts based on some special criteria to improve further processing. The purpose of slicing was at first to facilitate debugging, then, with the emergence of new algorithms and techniques, it became helpful in many aspects of the software development lifecycle (Xu et al. 2005): software testing, software maintenance, program comprehension (Harman and Hierons 2001), etc. Recently, slicing has been used as novel approach for detecting automatically vulnerable lines of code (Fu and Tantithamthavorn 2022; Hin et al. 2022; Yu-Tao et al. 2023).

Detecting vulnerabilities is a hard task, and giving the developers a slice of the original file (for example built on the seed identified by a static analyzer or a machine learning algorithm) should help to find them. It seems obvious: a sliced program is by construction smaller than the original program and thus finding something by inspection should be easier as well. As we shall see, the obvious might not necessarily be always true.

To answer this question, we performed a multi-year experiment with $n=236$ MSc students reviewing four large real-life Java files from Apache Tomcat with a CVE among the most common Top 10 OWASP 2017^1^ considering the four groups A1(Injection), A5(Broken Access Control), A6 (Security Misconfiguration) and A7 (Cross-Site Scripting). For simplicity, we refer to the four vulnerability types as path traversal (A5-A6), user injection (A1), XSS (A7), DoS (A5-A6). A combination of original and sliced files was presented to each participant to obtain an orthogonal balanced design. The participants submitted the line numbers that they identified as vulnerable for each project.

Vulnerable fragments are typically small and involve few lines in otherwise large files (e.g. Dashevskyi et al. 2018; Bao et al. 2022) and hence having a formal criteria to match the lines identified by the inspector and the lines identified by the security expert is critical. While several metrics (e.g. EXAM, Recall@10, etc.) exist to measure the *(many) lines proposed by a tool* Li and Zhang (2017), Li et al. (2019), Li et al. (2021), Sohn and Yoo (2017), to the best of our knowledge, no formal method exists for the *(few) lines identified by a human*. Only qualitative criteria are reported in the literature on human studies on code reviews (e.g. Braz et al. 2022). On the one hand, choosing the participants’ answers to exactly match the ground truth as narrowly identified by a security expert would dramatically reduce the number of correct responses even if the participants comprehended the program and essentially found the vulnerability. On the other hand, accepting as correct all lines potentially related would lead to overoptimistic results and make us conclude that slicing always works. The solution to this challenge is not uniformly agreed upon in program comprehension experiments. We propose in this paper the first such formal metric based on the intuition of the git diff context: a line in the ground truth is considered found if the code inspector suggested a line at distance $\delta$ (=0, 1,...3, ...) from the former.

We analyzed the data collected for two different values of $\delta$-neighborhood: (i) $\delta = 0$ to describe the case of exactly matching the ground truth vulnerable lines, (ii) and $\delta = 3$ to represent the scenario of identifying the vulnerable line relying on the value of the git diff command. For both cases we observed that slicing has a statistically significant effect in finding some lines of code related to the vulnerability as opposed to finding no related lines. However, for the case of $\delta = 0$ by using the same methodology used by the Food and Drug Administration (FDA) and the European Medicines Agency (EMA) for establishing the bio-equivalence between a branded version of a drug and its generic version, we also found that, once an inspector found something, being shown a slice is statistically equivalent to being shown the original file (the obvious is not always true). Contrarily, when considering $\delta = 3$, the two sample groups (original files vs sliced files) are not statistically equivalent, and the number of TPs for the slice treatment is larger than the number of TPs for the original treatment. This finding is in line with the common expectation as less line of code should make it easier to identify the vulnerabilities. Yet, using Master students as participants is a potential threat to the generalizability of the results. Therefore, more experiment are needed with experienced developers.

### Data availability statement

The replication package is available on zenodo at this link: https://doi.org/10.5281/zenodo.15022147. It contains the raw data of the participant’s responses, the reviewers may check the results. The tool used to create the slices is available on GitHub at this link: https://github.com/standash/foss-vuln-tracker/tree/master/repoman.

## Background and related work

### Developers vs. Security

Several studies proposed and improved tools to support developers in vulnerability detection (Ayewah and Pugh 2008; Ayewah et al. 2008; Smith et al. 2015). Smith et al. (2018) reports an exploratory study with novice and experienced software developers, highlighting strategic techniques to successfully detect vulnerabilities, and help developers identify them through static analysis tools. Developers still find these tools confusing and are still not widely adopted (Tahaei and Vaniea 2019).

Studies on developer-centered security have attempted to adapt Human-Computer Interaction methodologies and well-established usable security measures to software development, addressing developers’ needs (Green and Smith 2016; Pieczul et al. 2017; Wurster and Van Oorschot 2008). Naiakshina et al. (2017, 2018) assigned 40 Computer Science students into two groups and they received two different task descriptions: one did not mention security, the other requested to implement a security solution. The students in the first group did not store passwords securely.

Besides using tools for the identification of vulnerabilities, organizations are adopting modern code inspection techniques (Cohen 2010) trying to reduce the time spent on code inspection. Across companies (Bacchelli and Bird 2013; Sadowski et al. 2018), and community-driven projects (Rigby and Bird 2013; Rigby et al. 2014) modern code inspection or change-based inspection (Baum et al. 2016) is widely used. However, Tahaei et al.’s survey (2019) shows that code inspection is less studied in developer-centered security: only one code inspection study is reported (Edmundson et al. 2013). Only recently, some more studies have been performed. We discuss them in the next subsection.

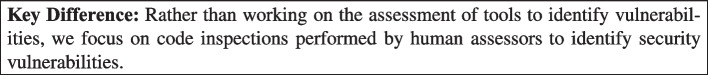



### Code Inspection

Code inspections have received increasing attention in the past years. Kollanus and Koskinen (2009) shows several empirical studies on code inspection that have been conducted over the years. The survey reports the results on 153 articles with a high impact publication from 1980 to 2008. The authors claim that the real issue with code inspection is its weak implementation in the software industry, and they suggest focusing more on empirical studies to understand why there is a lack of implementation and management of inspections in practice.

Indeed, relatively few studies within our related work search investigated the implementation and management of inspections in practice. Empirical studies are needed especially to solve these issues. Such gained body of knowledge can create a good basis for further studies.

Recently, there have been some studies on checklists for code inspections. Rong et al. (2012) conducted a study with students, and they claim that checklists helped the participants during code inspection. In the study (Chong et al. 2021), the students were able to anticipate potential defects and create relatively good code inspection checklists. Gonçalves et al. (2020) explored whether inspection checklists and guidance improve code inspection performances.

A study conducted by Braz et al. (2022), analyzes both experimental designs: *(i)* asking for secure coding practices, and *(ii)* providing checklists. A total of 150 developers took part in the experiment online with three different treatments. In the baseline treatment, the participants performed a code inspection without any special instructions. In the second treatment, the participants were informed to perform the inspection from a security perspective. The last treatment is similar to the second one with the addition of security checklists provided to the participants. The results obtained show that when asked to focus on the security perspective, the probability of vulnerability detection increases. However, no significant results have been obtained regarding the use of security checklists.

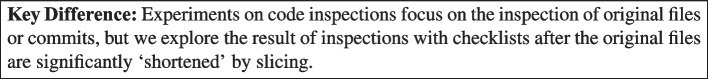



### Slicing and Program Comprehension

From the perspective of supporting debugging, several studies proposed program slicing as a fault localization technique (Ang et al. 2017) and different algorithms have been proposed to compute slices. They vary from Weiser’s syntax-preserving static slicing to amorphous slicing which is not syntax-preserving. The different algorithms can be based on data-flow or information-flow graphs (Xu et al. 2005).

In terms of effectiveness, Wong et al.’s survey (2016) compared slicing techniques to fault localization techniques highlighting their weaknesses and strengths in the identification failures. Also, Reis et al. (2019) argued that dynamic slicing combined with spectrum-based fault localization increases the performances in debugging.

In a recent large-scale study, Soremekun et al. (2021) analyzed 457 bugs (369 single faults and 88 multiple faults) in 46 open source C programs, to compare the effectiveness of statistical fault localization against dynamic slicing. They concluded that dynamic slicing is more effective for programs with single faults. However, this study was purely automated, using proxy measures for attention, and did not involve human participants.

Moreover slicing program has been used in recent approaches for detecting vulnerable lines of code (Salimi and Kharrazi 2022; Fu and Tantithamthavorn 2022; Hin et al. 2022; Yu-Tao et al. 2023).

  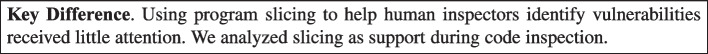


### Experimental Methods with Developers

Controlled experiments are often used to empirically evaluate and study new techniques and approaches. Over the years there have been papers showing experimental designs to study new solutions to support developers on debugging and fault localization (Böhme et al. 2017; Beller et al. 2018; Zhou et al. 2021; Alaboudi and LaToza 2020).

Controlled experiments are one type among the different empirical studies (e.g. case studies, surveys, etc.), and to conduct them, the researchers must navigate a large space for design decisions.

Several studies focus on giving guidelines when designing a controlled experiment for supporting program comprehension (Di Penta et al. 2007; Dunsmore and Roper 2000; Rajlich and Cowan 1997; Rajlich et al. 1994; Feigenspan et al. 2011; Siegmund and Schumann 2015). Among others, these guidelines suggest important rules such as: *(i)* identify the key variables to measure, *(ii)* select the appropriate subject population, *(iii)* standardize the experimental design format to allow replication, and *(iv)* provide procedures and criteria for packaging the materials and results to facilitate replication.

Among the issues highlighted, Siegmund and Schumann (2015) discuss which parameters should be taken into account while designing a controlled experiment, and other studies show which measures should be considered (Feigenspan et al. 2011; Rajlich and Cowan 1997). In particular, Sulír (2019) claim that only 15% of the studies use real tasks from issue trackers, and sometimes they are modified to fit the purpose.

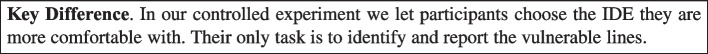



## Research methodology

In this section, we introduce our research questions and detail our experimental design.

### Research questions

We structure our study around three research questions. We aim to define a methodological design of experiments to establish a new metric to evaluate fault localization by humans. The experiment on eye-tracking by Uwano et al. (2006) shows that code inspectors, after a first scan of the entire code, mainly focus on some portions of the code but still fluctuate around the area. Irrelevant fluctuations might then be reflected in the answers. For example, an inspector might mark the beginning and the end of an if-block without marking the if statement itself. Still, that would be a clear indication of where the problem was. Hence, we state our first research question as follows:

**RQ1**: What does it mean for a human inspector to identify a vulnerable fragment in a file spanning a thousand lines of code?

If slicing can help find vulnerabilities, then from a company’s perspective it could make sense to invest resources (training of developers, consultants, integration of toolchains, etc.) to develop and deploy a plug-in on a developer’s IDE. Indeed, Hirsch and Hofer (2021) suggest that programmers often use IDE built-in tools for debugging, therefore, before investments into tools integration are made, evidence of slicing supporting code inspectors would be useful. Therefore, we state our second research question as follows:

**RQ2:** Does a method-level slicing program help code inspectors identify more vulnerable code lines than inspecting the full source code?

Finally, since vulnerabilities are widely different, we also wanted to investigate whether some vulnerability types are easier to detect during code inspection. We formulate our third and last research question as follows:

**RQ3:** Which vulnerabilities are better identified by method-level slicing?

The choice of a measure of success is critical to provide a result that is informative for the concrete experiment at hand. Precision and recall as ratios are informative when used in the framework of ML (Chakraborty et al. 2022) or information retrieval of vulnerabilities (Pashchenko et al. 2022; Cadariu et al. 2015) where both numerator and denominator are large numbers. In a code review or inspection set up each participant has to report a handful of lines of code. For example in CVE-2012-2733 (See further in Table 4) there are three vulnerable lines to be identified (459, 462, 463). Thus, a difference between 100% recall to a 66% recall might correspond to a difference in the identification of 1 line of code. Precision and recall would thus provide misleading information.

The same considerations apply to the Jaccard index or other metrics of overlap between images (e.g. Crum et al. 2006) that considers the ratio between the numbers of several hundred pixels or voxels (3D volume elements). A participant reporting lines 459-463 for the mentioned example for CVE-2012-2733 would score a Jaccard index of 60% (3 correct lines overlapping with the 5 suggested) when any developer reviewing the result would consider the vulnerability to have been correctly identified.

Other metrics such as top-1, and EXAM focus more on evaluating the tools’ performance in fault localization, which is different from the goal of our study as we want to evaluate the humans in identifying the vulnerable source lines of code. Among the existing techniques, the most relevant ones are EXAM, Top-N, Mean First Rank (MFR), and Mean Average Rank (MAR) which are summarized in Table 1. We highlight the difference of these metrics from our custom metric whose input is a human, and not a tool.

**Table 1:** Metrics for fault localization

Metric	Definition	Input
EXAM	The percentage of code that a developer has to examine until the first fault is located (Meng et al. 2022)	Tool
Top-N	It is the number of faulty codes for which the fault localization successfully identified the fault within the top N positions (Meng et al. 2022)	Tool
MFR	The mean of the highest faulty statement’s rank of each fault (Meng et al. 2022)	Tool
MAR	The mean of the average rank of all the faults of a project (Meng et al. 2022)	Tool
$TP_{\delta }$	Each vulnerable line which is distant at most $\delta$-lines from a line suggested by a participant (this paper)	Human

We summarize the most relevant existing techniques for fault localization and we compare them with our own metric (last row)

The techniques reported in Table 1 have been used in previous fault localization studies Li and Zhang (2017), Li et al. (2019), Li et al. (2021), Sohn and Yoo (2017). They have also been largely used to evaluate the most common fault localization techniques such as Tarantula (Jones and Harrold 2005), and Ochiai (Abreu et al. 2006). However these metrics are tailored to evaluate tool techniques, and they do not apply to our study as Ochiai (Abreu et al. 2006) requires more runs as it calculates the fraction of the number of times a faulty line is executed over the total number of runs. In our case, we only have a human manually inspecting a piece of source code, and we want to evaluate their capability of inspecting the vulnerability.

### Measuring True Positives identified by Humans

As we mentioned in Section 1, our first challenge was choosing a neighborhood $\delta$. Choosing it too big would lead to unrealistic results. Too small could mean counting too many false positives, even if, for practical purposes, the participants essentially identified the code region where the vulnerability was.

**Fig. 1:**
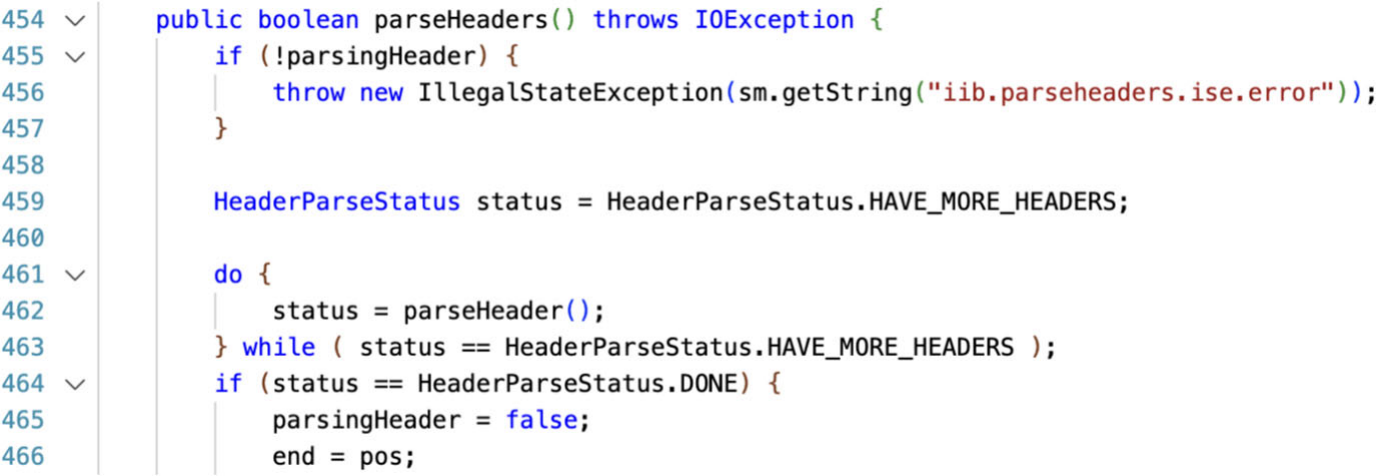
Extract of code with the vulnerable lines (459, 462, 463) from CVE-2012-2733 (Denial of Service)

Consider the following concrete example from CVE-2012-2733 where Fig. 1 illustrates the actual lines of code.

The security experts identified three lines as the lines responsible for the vulnerability and one participant suggested other four different lines. The set *GT* is the ground truth, the set of vulnerable lines as assessed by a security expert, the set *Participant* is populated by a participant’s answer.

1 $$\begin{aligned} GT= &  \{ 459, 462, 463 \} \end{aligned}$$

2 $$\begin{aligned} Participant= &  \{ \underline{457}, \underline{458}, \underline{459}, \underline{500} \} \end{aligned}$$

The key problem is devising a reasonable evaluation of the lines 457, 458 because they are not the actual vulnerable lines (so they should not count as truly true positive), but they are also extremely close to the line 459 (identified by the security experts). In this case, among almost a thousand lines of code, the participant identified the vulnerability region, therefore, it should not be considered a false positive.

#### Traditional metrics.

If we use precision, recall, or the Jaccard Index we would have

3 $$\begin{aligned} P=GT\cap Participant/Participant = 1/4=25\% \end{aligned}$$

4 $$\begin{aligned} R=GT\cap Participant/GT=1/3=33\% \end{aligned}$$

5 $$\begin{aligned} J=GT\cap Participant/GT\cup Participant=1/6=16\% \end{aligned}$$

Those numbers are far from the intuitive understanding of what is concretely happening in the inspection of a file with over 800 lines of code. The participant identified the region: 1 clear TP, essentially 2 out of 3 visible in a git context, and 1 clear FP. Precision, recall, and the Jaccard index are *summary* indicators that they are reliable when the numerator and denominator are large numbers derived from large samples. However, this is not applicable in our case: the difference in +1 or -1 line of code makes those summary indicators widely swing.

#### A local but still coarse grained metric

. As an initial attempt, we created a $\delta$-neighborhood by adding all lines away from $\delta$ from the Ground Truth by $\delta$ line numbers.

The Ground truth would have thus been $\text{ neighbor}_{\delta }\left( GT\right)$. This made it easier to test potential TP ($Participant\cap \text{ neighbor}_{\delta }\left( GT\right)$) but we realized that it would have inflated the number of potential positives up to $2\cdot \delta \cdot |GT|$ and also generated a large number of FN. The FP rate would have been instead meaningful since it indicates lines that are really far off from the target ($Participant\setminus \text{ neighbor}_{\delta }\left( GT\right)$).

In our example the 3-neighborhood would have been the interval of ground truth lines +/- 3 lines. In this particular example, the 3-neighborhood interval is 456, 457, 458, 459, 460, 461, 462, 463, 464, 465, 466. Any participant’s answer line falling in this interval would have been considered valid as identification for the vulnerable lines of the ground truth.

#### Our proposed metric

Therefore, we designed a more precise classification of the TP that would also avoid inflating the FN.

$$TP_\delta =$$
all GT lines whose $\delta$-neighborhood intersect the lines suggested by a participant $TP_\delta =\left| \left\{ \ell \in GT: Participant \cap \text{ neighbor}_{\delta }\left( \ell \right) \right\} \right|$
$$FP_\delta =$$
all remaining lines suggested by a participant that are not in the $\delta$-neighborhood of the entire GT.
$$FN_\delta =$$
all remaining lines in GT that are not TPs.

The key idea is that we count as TP the lines that identify a region. If the region is the same, then we should count all lines pointing to the same region of a vulnerable line as a single TP. The more vulnerable lines fell into the region identified by the participants, the better but this should not needlessly inflate the number of either TPs or FPs.

Using again the concrete example from CVE-2012-2733, with $GT = \{459, 462,463\}$ and $Participant = \{457,458,459,500\}$, in Table 2 we show the result that we would obtain with $\delta =0$ and $\delta =3$.

**Table 2:** Illustration metrics application

	$\delta = 0$			$\delta = 3$		
	GT lines					
P lines	459	462	463	459	462	463
457	✗	✗	✗	✓	✗	✗
458	✗	✗	✗	✓	✗	✗
459	✓	✗	✗	✓	✓	✗
500	✗	✗	✗	✗	✗	✗
	Metric					
	TP	FP	FN	TP	FP	FN
Total	1	3	2	2	1	1

We show the concrete example of CVE-2012-2733. Each row corresponds to a line from the $Participant = \{457,458,459,500\}$ variable, and each column corresponds to a line from the $GT = \{459,462,463\}$ variable. On the left side of the table, we show the results for $\delta = 0$, and on the right side the results for $\delta = 3$. We indicate with ✓if a P line falls in the $\delta$-neighbor of a GT line, otherwise we use the symbol ✗. At the bottom of the table, we report the final result for the metrics TP, FP, and FN

For the case of $\delta = 0$ we have $TP_{\delta =0} = 1$ for the line 459, which exactly matches the ground truth line, $FP_{\delta =0} = 3$ for the lines $\{457,458,500\}$ as they do not match any of the ground truth lines, and $FN_{\delta =0} = 2$ on the remaining missed lines $\{462,463\}$ in the GT.

We can then compute the $\delta =3$ neighborhood of the ground truth *GT* for the three lines indicated by the expert.

6 $$\begin{aligned} \text{ neighbor}_{3}\left( 459\right)= &  \left\{ 456, \underline{457}, \underline{458}, \underline{{\textbf {459}}}, 460, 461, 462\right\} \end{aligned}$$

7 $$\begin{aligned} \text{ neighbor}_{3}\left( 462\right)= &  \left\{ \underline{459}, 460, 461, {\textbf {462}}, 463, 464, 465\right\} \end{aligned}$$

8 $$\begin{aligned} \text{ neighbor}_{3}\left( 463\right)= &  \left\{ 460, 461, 462, {\textbf {463}}, 464, 465, 466\right\} \end{aligned}$$

To visualize the result of the computation we mark with boldface the lines of the ground truth and underline the lines identified by the participant. For the case of $\delta = 3$, we have $TP_{\delta =3}=2$ for the lines $\{457, 458, 459 \}$ which falls into the 3-neighborhood of the lines $\{459, 462\}$, $FP_{\delta =3}=1$ corresponding to line $\{ 500 \}$ which fall in none of $\text{ neighbor}_{3}\left( 459\right)$, $\text{ neighbor}_{3}\left( 462\right)$, or $\text{ neighbor}_{3}\left( 463\right)$ and $FN_3=1$ on the remaining missed line $\{463\}$ in the GT. Moreover, since line $\{459\}$ falls into both the 3-neighborhood of the lines $\{459, 462\}$, we count it as one single *TP*.

### Experimental design

**Fig. 2:**
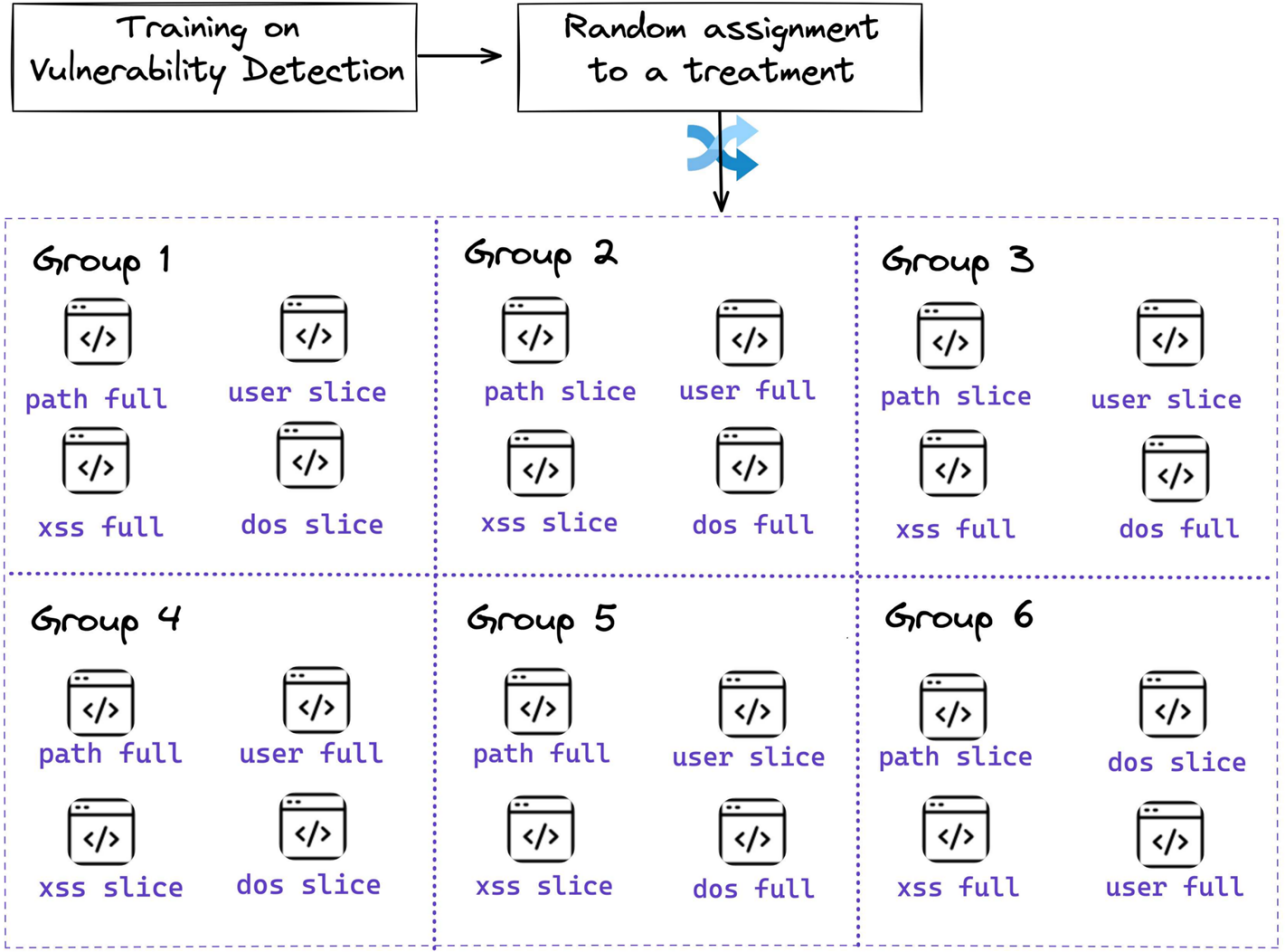
Steps of our experiment

Figure 2 summarizes the experimental protocol that we designed.

If we consider having *k* factors, $\ell$ levels, in a full factorial design we test all the possible conditions which are equal to $\ell ^{k}$. Even if this design reveals all the combinations, it is time-consuming and requires many subjects. In our scenario we compare sliced files and original files, therefore, we have $\ell =2$ and $k=4$ factors, one for each vulnerability type (path, user, XSS, DoS). Therefore, our scenario would yield 16 different conditions. As our goal is to compare subjects that did not identify the vulnerable lines vs subjects that identified at least some vulnerable lines, this would have required two additional conditions for each scenario. Such an experimental setup would require too many conditions (*n*) for an experiment with human subjects because obtaining some statistical confidence would require making sure that at least 5 and possibly 15 participants are assigned to each condition (at least $n=2^{4}\times 2 \times 5-15=145$).

An alternative is to use crossover designs, common in software engineering experimentation. Since subjects apply more than one treatment, this kind of experiment requires fewer participants and controls the variability among subjects. Vegas et al. (2015) argue that crossover experiments can yield valid results, therefore, we decided to design a balanced crossover experiment. In our experiment, each treatment differs in the pair *(Vulnerability, Type of Code)*. Hence, we opted for the orthogonal array experiment, where the number of treatments is then $(k-1)\ell$. Massacci et al. (2024) formally analyzed this particular form of design which makes possible to control for learning effect. This means that the experimental unit is the pair *(participant, vulnerability scenario)*. Therefore, for each participant, there will be two data points for the slice intervention and two data points for the full intervention (a total of four data pairs for each participant), as discussed by Massacci et al. (2024). Therefore, if we consider the case of three slice interventions in which no vulnerable lines were found, two data points could come from the same student, the third one from a different student, or all three data points could come from three different students.

Therefore, we designed 6 different groups, each of them to be tested for the cases when some vulnerable lines have and have not been found. Each subject is randomly assigned to execute four different assessments, each described in a square in Fig. 2.

We gave each participant an equal share of full and slice source code for two reasons. *(i)* Slice intervention reduces the time of code inspection, Jaber et al. (2020) show that program slicing techniques reduce the time of code inspection process. Therefore, assigning the participants to only full treatments, or slice treatments would have led to an unbalanced experiment design, as inspecting a slice of code takes less time than inspecting the full source code. *(ii)* Moreover, the experimental time is fixed and is the same for all the participants. Splitting the participants into two groups (full treatment and slice treatment) would lead to unfair results as inspecting a slice of code takes less time and the participants would have more time to inspect the code and find more vulnerabilities.

### Experimental protocol

#### Selection of Participants

Participants are by convenience sampling as MSc Students attending Class 1 at University 1, and Class 2 at University 2. Both classes were elective security courses. The objectives of both classes included performing and designing experiments of attack and defense to analyze techniques to improve code inspection. Students had to analyze and present the results to the class.

#### Training

The first part of the experiment consists of a training phase on the detection of vulnerabilities in source code. Its purpose is to fill the security knowledge gaps of the participants since not all of them have a strong cybersecurity background.

The duration of the training is an important experiment parameter but there is not enough consensus in the literature regarding the length of the training. In the field of threat analysis and security requirements, training activities sometimes last several hours (Scandariato et al. 215; Wuyts et al. 2014) or even days (Tuma and Scandariato 2018). In the domain of APR tools, Chong et al. (2021) included training of several weekly lectures of 60 minutes. In contrast, Tao et al. (2014) designed only a ten minute tutorial. Other studies planned an introduction, instruction, or a tutorial for their participants, however, these works do not mention the duration of their training phase (Naiakshina et al. 2017; Cambronero et al. 2019; Zhang et al. 2022; Fry et al. 2012). Other works do not mention any training for their participants (Rong et al. 2012; Gonçalves et al. 2020).

We based our training design on previous studies where professionals were involved, and the minimum training duration for these works was 1.5 hours (Allodi et al. 2020; Gramatica et al. 2015; Tuma et al. 2021). In the industry reality, this is the average duration for a typical session of professional training phases^2^. Moreover, the survey reported by Kollanus and Koskinen (2009) also report that “overview meetings” are frequently used in software inspection publications. The term *meeting* does not suggest presentations of 10 or 30 minutes, nor day-long events, therefore, we concluded that the duration of 1.5 hours was reasonable.

**Fig. 3:**
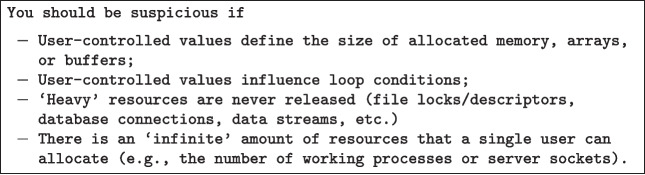
Denial of Service: how to find it checklist

The training was performed through the support of the platform Qualtrics. We created a survey that has two goals: *(i)* to register the participants and assign them to the different groups and *(ii)* show five different videos that we recorded. The first video is a general introduction to security vulnerabilities, the following four videos cover in detail each vulnerability type that we selected for the experiment: user injection, XSS, path traversal, and denial of service. The participants’ objectives achieved at the end of the training are gaining knowledge about *(i)* 4 specific vulnerability types, and *(ii)* identifying them into programming code also supported by simple checklists. In particular, to offset potential issues due to individual usages of checklist (see e.g. Braz et al. 2022), each training section was concluded with a simple checklist that participants could use. We report the example of denial of service in Fig. 3.

The training Qualtrics survey was released to the students one day before the experiment, and it was accessible until two hours before the start of the experiment, therefore, they had 24 hours in total to complete and submit the survey. One hour and a half is the total duration of the five training videos.

Once the students successfully submitted the survey, they received an email with a link to a Google Drive folder containing all the training material such as lecture videos, digital copies of lecture slides, technical documentation, etc. to be consulted at any time, also during the execution of the experiment.

#### Randomization

We used the training Qualtrics survey system to randomly assign the students to one of the six treatments. The treatments differ in the pair *(Type of Vulnerability, Type of Code)*. We collected the variables *Assignment to a Vulnerability Treatment* and *Assignment to a Type of Code Treatment*. The first describes the assignment of each participant to a type of vulnerability, given the variable assignment to a type of code. The second describes the assignment of each participant to a type of code: *(i)* slice of code, or *(ii)* original file code.

**Experiment**. The participants performed the experiment in a laboratory located on campus, and they had a maximum of 100 minutes to complete it (which allowed, on average, 25 minutes for each vulnerability). The participants were separated into different rooms according to their treatment group to avoid spillover effects. Each room was supervised by an experimenter whose role does not go beyond the supervision of the room and the technical support, s/he couldn’t reply to questions regarding the solution of the correct vulnerable lines. S/he however controlled that participants did not talk to each other. We assumed that the participants would not have used the internet or chat messages to talk to each other. We discuss this issue further in the threat to validity.

The participants were tasked with identifying the vulnerabilities in the code files provided through a link to a Google Drive folder. Each treatment had a link to a different folder. The code files provided to the students were simply non-runnable Java code. Once the participants downloaded the four files, they could open the files, and inspect the code using the IDE they were more comfortable with. The participants submitted their answers through the Qualtrics survey by writing the number of the vulnerable lines identified. Moreover, to have a deeper level of analysis and understanding of the answer reported, and to avoid doubts about the reliability of the results, the participants were asked to explain why they deemed certain lines as vulnerable. The participants could follow the recommendations to perform code inspection provided during the training (e.g. see Fig. 3).

**Table 3:** Experimental Variables

Name	Description	Operationalize
*Independent variables (design)*		
Assignment to a Vulnerability Treatment	Random assignments of the participants to a vulnerability (path traversal, user injection, XSS, DoS).	Nominal (A)
Assignment to a Type of Code Treatment	Random assignments of the participants to a type of code (full vs. slice).	Nominal (A)
*Background variables*		
Knowledge of Java	Self-reported concrete experience on Java.	Ordinal scale (B)
Knowledge of Security Vulnerabilities	Self-reported concrete experience on vulnerability assessment.	Ordinal scale (B)
Knowledge of SW Development IDE	Self-reported concrete experience on software development IDE.	Ordinal scale (B)
*Dependent variables*		
Vulnerable Lines Found	Number of correct vulnerable lines identified by the participants.	Ratio (C)

**(A)** Automatically performed by the Qualtrics submission tool; **(B)** Multiple choice: no experience, attended a tutorial, attended a course, company internship, professional practice; **(C)** Text insertion of the interval of the vulnerable lines

### Measurement plan

Table 3 shows the independent and dependent variables of the experiment. The survey used to collect the data included three parts: the *Background* section included questions on knowledge of Java, knowledge of security vulnerabilities, and years of experience in coding, and code inspection; the *Experiment* section was used to collect the experimental data;

#### Training Compliance

Initially the format of the experiment was slightly different as the training was conducted in person. Due to the Covid pandemic, we were forced to switch the experiment to an online format. To assure the quality of the data we collected quality control measures as *Time spent on the video*, *Perception* questions on the usefulness of the training material, difficulty of the questions, and an open question to provide additional comments to improve the experiment process. Since the purpose of the training is to train participants on security vulnerabilities detection into source code, we collected the variable *Time spent on the video* to check whether the students watched the training video/material. To guarantee the quality of the data we decided to keep the perception questions in the survey also after the end of the pandemic period.

#### Experimental Background

The objective is to ascertain whether the participants have any experience in different contexts (e.g. University projects, personal developed projects, or professional experience) that might impact the result of the experiments. We collected the variables *Knowledge of Java* and *Knowledge of Security Vulnerabilities* asking if they attended a tutorial, a full course, have done vulnerability assessment in an internship, or have true professional expertise.

#### Experimental Outcome

Moreover, to answer to RQ1 and RQ2 we need to know the number of correct vulnerable lines identified by the participants, which is measured through the variable *Vulnerable Lines Found*. We collected the participants’ responses, and we implemented a Python script to count the number of correct vulnerable lines that have been reported. A vulnerable line is considered correct if it is included in the ground truth interval (see Section 3.1).

### Analysis procedure

#### Statistical Tests

To answer our research questions we want to determine whether there is a statistically different distribution between the slicing and control. As common to many computer science experiments and in particular on the analysis of security vulnerabilities which are rare and hard to find, we expected to have an excess of zeros. In other words, we expected our dataset to be divided between those who “found nothing” and those who “found something”. We, therefore, followed Lachenbruch’s statistical recommendations to correctly analyze datasets with an excess of zeros (Lachenbruch 2002) and split the analysis into two parts: a discrete test (a $\chi ^2$ contingency table test) to test the differences between those that “found something” and those that “found nothing” and a test for an ordinal or continuous variable (t-test, Mann-Withney U test, or Kruskal-Wallis H test) to distinguish among those that found something.

As we considered the possibility of no effect, we used TOST (Two One-Sided Tests) as a test of equivalence which was initially proposed by Schuirmann (1981) and is widely used in pharmacological and food sciences to check whether the two treatments are equivalent within a specified range (either as an additive constant or as a ratio) (Food and Drug Administration 2001; Meyners 2012). For the US Food and Drug Administration and the European Medicine Agency, two drugs are considered equivalent if their respective distributions *x* and *y* are such that $x \cdot \rho < y$ (one-sided test) and $y < x \cdot 1/\rho$ (the second one-sided test) for $\rho = 0.8$. The results of the two tests are conservatively combined by taking the maximum of the two *p*-values. The underlying directional test can be either the Mann-Whitney U (MWU) test or the t-test depending on the conditions at hand. In our study, to answer RQ2 and RQ3, we used MWU test. The null hypothesis for RQ2 is that there is no difference between the two groups original files and slice files. Similar for RQ3 but considering one type of vulnerability at a time. For both research questions, we set as p-value (threshold) 0.05. To be conservative, we adopt the value of $\rho$ used by the FDA and EMA.

#### Measures of Success

We proposed an innovative solution based on the intersection between the $\delta$-neighborhood (in terms of lines of code) of the vulnerable lines and the lines identified by the participant. Given the criticality of this issue, we dedicate a full section to it (§3.1).

### Compliance with ethical standards

The students received a bonus point for the course for participating in the experiment. The ethical procedure was followed and it determined that a full ethical review was not necessary. In particular, this was determined because (i) upfront, opt-in consent was asked, (ii) no personal or sensitive information was involved, (iii) it did not pose potential risks to either participants or researchers, (iv) the confidentiality of the participants was guaranteed by collecting data by GDPR compliant tool and removing their details before processing the data for the analysis, (v) the incentives to participate were minimal, (vi) and the participants were minimally deceived and thoroughly debriefed afterward (they actually had full access to the anonymized data). There was no monetary compensation, and the participants received compensation in terms of a coursework bonus. Such value was minimal (less than 2% of the final grade), and the participants could deny the consent, and still obtain the participation bonus. The personal details were only collected to grant the coursework bonus.

## Experimental objects

The experimental objects consist of the slicing tool to generate the slice source codes and the dataset of the vulnerabilities selected.

### Slicing algorithm

In practice, the seed would need to be identified first and a wrong localization might change the result. To control for this factor, we provided the perfect seed lines to the slicing algorithm by a manual review of the diff between a vulnerable method and its security fixing commit as suggested in the literature (Dashevskyi et al. 2018; Salimi and Kharrazi 2022; Bui et al. 2024). In this way we can focus on studying whether the output of a method-level slicing algorithm, improves participants’ effectiveness in identifying vulnerabilities. The choice of the slicing algorithm may also influence the results: some algorithms generate larger but more accurate slices (Salimi and Kharrazi 2022), others, such as intra-procedural thin slicing, have less accurate but smaller slices (Sridharan et al. 2007; Dashevskyi et al. 2018). The former is more suitable for automated processing, the latter for human reading, and hence our choice.

To generate the slices from the original source codes, we used the slicing tool implemented by Dashevskyi et al. (2018). Compared to the original slicing algorithm by Weiser (1984) that requires the resulting slices to be executable, the adaption proposed by Dashevskyi et al. (2018) does not have this requirement, they have deliberately sacrificed the precision in favor of scalability.

The authors did not use a traditional program slicing (Weiser 1981) for extracting vulnerable code as *‘traditional slices often grow too large’* (Sridharan et al. 2007). Dashevskyi et al. (2018), Dashevskyi et al. (2018) implemented a generalized intra-procedural version of *thin slicing* introduced by Sridharan et al. (2007), for finding relevant vulnerable source code statements with the SZZ approach adapted from Śliwerski (2005) for tracking the vulnerable code back to its introduction. The resulting slices are limited to intra-procedural boundaries since the analysis of the vulnerability fixes proposed in Dashevskyi et al. (2018) suggests that security fixes are rather ‘local’. In their case, the lines modified during a vulnerability fix are *seeds*, and, similarly, to the study from Sridharan et al. (2007), a slice includes a set of *producer statements* for these seeds. A producer statement is an assignment of a value to a certain variable. To identify simple dependencies between statements they look for relevance relations between variables in them. They also include a set of *explainer statements* that are relevant to the *seeds*. An explainer statement can be a statement that represents the expressions in the condition branches under which a producer statement will be executed (control flow). Otherwise, an explainer statement can correspond to a method call that has a parameter to which a value flows from a producer statement (sink).

An alternative would be to use JoanAudit Thomé et al. (2017) Thomé et al. (2018) which is a static analysis tool for auditing Web applications and Web services for common injection vulnerabilities. It also applies program slicing technique and generates an HTML report to guide security auditors audit the source code. The tool by itself works on entire projects and therefore produce a large report. Since our study focuses in understanding whether a slice helps the users’ task of identifying vulnerabilities in a single file we used the thin-slicing tool for building a slice from a seed proposed by Dashevskyi et al. (2018) which is generalized intraprocedural version of thin slicing introduced by Sridharan et al. (2007), which improves over the notion of statement relevance of the original slicing by Weiser and avoid the collection of more accurate, but potentially very large slices as would be generated by Thomé et al. (2017); Salimi and Kharrazi (2022)

The Algorithm 1 from Dashevskyi et al. (2018) is used for recursively finding a set of source code statements that are affected by the *Seeds* (a forward slice). Similarly the Algorithm 2 is used for recursively finding a set of source code statements that affect the *Seeds* (a backward slice).

**Algorithm 1:**
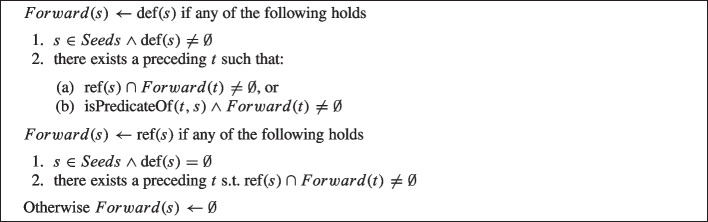
Forward Slices Computation

**Algorithm 2:**
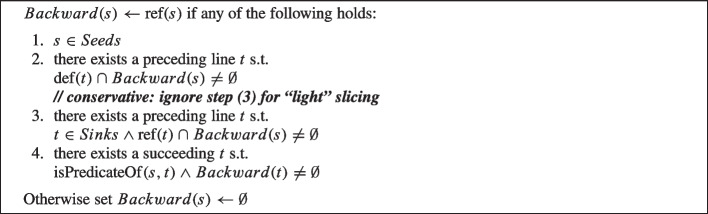
Backward Slices Computation

As a first step they start with the set of seed statements *Seeds* as the slicing criteria, where every criterion can be represented as a tuple $\langle s, V\rangle$ (similarly to Weiser’s slicing criterion Weiser 1984) where *s* is the seed statement, and *V* is the set of variables of interest in that statement: for each seed statement *s*, the set *V* consists of the variables which values are used in *s*.

Then, for every seed statement *s*, they iteratively identify other statements that contain relevant variables that are dependent on (Algorithm 1) or influence (Algorithm 2) the set of relevant variables *V* in *s*. The final slice includes all statements in the method for which there is at least one variable that is relevant to the seeds and, thus, will contain a union of statements returned by Algorithm 1 and Algorithm 2 ($Forward(Seed) \cup Backward(Seed)$).

To identify the relevant statements for the slices the algorithms make use of the following constructions.

#### Producer statements

“[...] statement *s* is a producer for statement *t* if *s* is part of a chain of assignments that computes and copies a value to *t*” (Sridharan et al. 2007). This is an assignment of a value to a certain variable: $t\in \text{ def }(s)$.

#### Explainer Control flow statements

the statements that represent the expressions in the condition branches under which a producer statement will be executed (this concept is taken from Sridharan et al. 2007 as well). A statement *s* is control dependent on a conditional expression *e* if *e* can affect whether *s* is executed. A statement *s* is flow dependent on a statement *t* if it reads from some variable *v* that is defined or changed at *t*, or there exists a control flow path from *t* to *s* on which *v* is not re-defined.

#### Explainer Sink statements

represents a statement that corresponds to a method call that has a parameter to which a value flows from a producer statement. Therefore, a statement *s* is a relevant sink of the statement *t* if *s* is a method call and *s* is flow-dependent upon *t*. In symbols $\text {ref}\!\left( {s}\right) \cap Forward(t)\ne \emptyset$ for forward slicing and similarly for backward slicing.

Further explanations are provided in the paper (Dashevskyi et al. 2018).

### Selection of vulnerability types

As our study is a multi-year study that we performed for the first time in 2017, we relied on the OWASP top ten 2017^3^ to select the security vulnerabilities. To remain consistent through the years and not change the experiment setup, we used the same rank through the years.

The type of vulnerabilities (and their corresponding CVEs) were selected based on several criteria: (i) simplicity, (ii) vulnerabilities due to lack of resources, and (iii) limit to vulnerabilities that can be identified by inspecting the program source files.

For example, there are many security vulnerabilities not affected by intervention but rather due to a lack of development resources (e.g. *A9: Using Components with Known Vulnerabilities*, or *A10: Insufficient Logging & Monitoring*). Others are not related to errors to be found in programs (e.g. *A6: Security Misconfiguration* for what concerns configuration files or *A3: Sensitive Data Exposure* where the data has not been encrypted but the program is otherwise perfectly “correct”). Other vulnerabilities are too complex for an experiment as they require specialized knowledge to understand the syntax, while conceptually they do not introduce a new type of vulnerability. For example *A4: XML External Entities (XXE)* and *A8: Insecure Deserialization* require already specialized skills from the developer in terms of XML processing or distributed data processing.

### Selection of concrete vulnerabilities

For the experiment, we selected four CVEs (shown in Table 4 Dashevskyi et al. 2018) for each vulnerability type we selected from OWASP top ten 2017: CVE-2008-2370 (Path Traversal), CVE-2009-0580 (Injection), CVE-2014-1904 (XSS), CVE-2012-2733 (DoS). The list of vulnerabilities and the vulnerable lines ground truth for the original files are reported in Table 4. We selected these vulnerabilities for simplicity as the lines affected are few, moreover, all the vulnerabilities are Apache Tomcat exploits, which is a popular technology and a known technology for the participants. Moreover, we decided to select the vulnerabilities from the same project because we wanted to avoid significant variations of coding styles and changes among the vulnerabilities, this increases the internal validity of the design. However, it poses some threats to the generalizability of the results. Information on the provided material can be found in the link to the replication package (Aurora Papotti and Fabio Massacci 2023).

We selected source codes coming from original repositories, therefore, we determined the vulnerable lines by inspecting the fixing commit of the developers in the project. According to the studies (Nguyen et al. 2016; Bao et al. 2022) the lines changed or deleted by developers may be considered responsible for the vulnerability, these lines are called *seeds* in the algorithm of Dashevskyi et al. (2018). This is also in line with the assumption *“EA2. Using Fix Locations as Root Cause Bug Diagnosis (aka “Fix Location”)”* in Soremekun et al. (2023): fix locations are used as substitute fault locations to determine the ground truth.

**Fig. 4:**
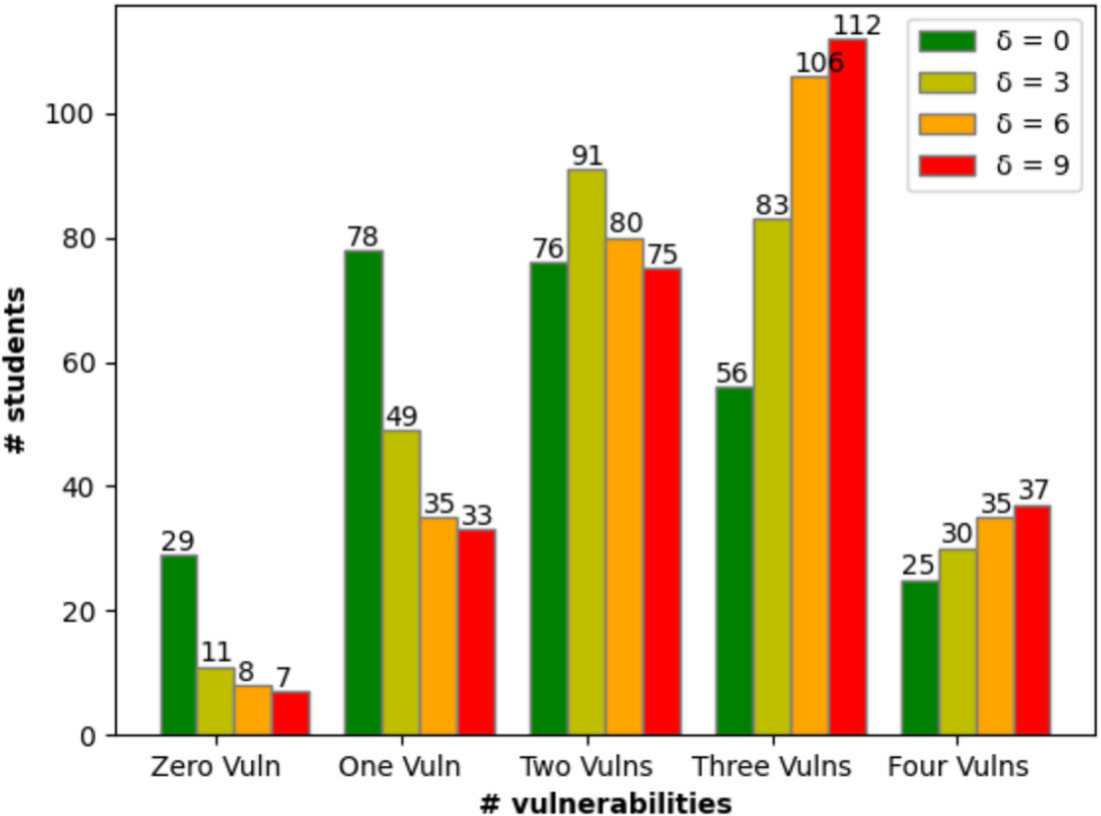
Number of vulnerabilities found by each participant for different values of $\delta$

Hence, we used the selection algorithm proposed by Dashevskyi et al. (2018), and with the help of two security researchers with several years of experience, we reviewed the fix commit and identified the vulnerable lines, which are reported in Table 4. We chose this algorithm because it does not require the build of the entire project, also our main contribution is not the choice of the slicing algorithm as we already provide the perfect seed lines to it (Fig. 4).

**Table 4:** Vulnerable Lines Security Vulnerabilities Dataset

	File Name	Vulnerable Lines
CVE-2008-2370	ApplicationContext.java	[368, 383...388, 432, 846, 853,
		854, 859, 860, 864]
CVE-2009-0580	MemoryRealm.java	[144, 147, 151, 287, 290]
CVE-2014-1904	FormTag.java	[441, 444, 450, 451]
CVE-2012-2733	InternalNioInputBuffer.java	[459, 462, 463]

## Results

In this section, we report the results of our investigation. In Section 5.1 we describe our population, in particular the background (e.g. knowledge of java and vulnerability assessment), and we provide a validation whether our population is a good sample to answer our research questions. Section 5.2 focuses on answering RQ1, instead in the sections 5.3 and  5.4 we answer respectively to RQ2 and R3, reporting the results both for $\delta = 0$ and $\delta = 3$. Moreover, when reporting these results, we decided to exclude the participants that declared to not have any experience with Java.

As we observed, both for $\delta = 0$ and $\delta = 3$, a large number of TPs equal zero (corresponding to cases in which the participants did not identify the vulnerability). Therefore, the choice of the combined test turned out to be appropriate. The statistical analysis was performed according to the choice of statistical tests explained in Section 3.6.

### Validation of participants data

A total of 264 Computer Science master’s students participated in the experiments which were collected across six years (from 2017 to 2023). Among them, 103 were enrolled in the course 1 from University 1, the others in the course 2 from University 2. As a population, this is consistent with several other studies (Naiakshina et al. 2017, 2018; Rong et al. 2012; Chong et al. 2021).

To validate their experience we looked first at their Java expertise and security expertise as collected during the Background phase (see 3.5). The scale values went from *“No experience with Java / Vulnerability Assessment”* to *“Strong experience with Java / Vulnerability Assessment through project performed outside University”*. This information is missing for 42 students to an experimenter’s error while collecting the data (data was optional).

Both groups from either University have written several programs in Java in hands-on labs or during short internships. There’s a slight difference in the Vulnerability Assessment’s experience: students from University 1 have a stronger experience due to a mandatory course in their study plan on Security Testing.

#### Knowledge of Java

Most of the participants from University 1 (42%) have written several programs in hands-on labs or during short internships. Some students (21%) have developed some significant projects, and others (21%) declared to have attended a course/tutorial. Finally, some students (15%) have attended only a couple of lectures. Several students from University 2 (42%) used Java as part of university projects and only 20% of the students reported having no experience. Some students (20%) said they developed several projects, and some (17%) developed Java projects outside the university context. As mentioned above, this data was missing for 42 participants (from University 1). As the population did not differ through the years we think that the reported participant background is very likely accurate despite the missing data points.

#### Knowledge of Vulnerability Assessment

Most of the students from University 1 have attended a course/tutorial on buffer overflow or software testing (48%), few have used vulnerability and network scanners (7%) during professional experiences, and some have used a fuzzer or vulnerability scanner in hands-on labs or during short internships (29%). Few students attended a course on hacking and security (16%). Most of the students from University 2 (49%) have no experience with vulnerability assessment; some performed vulnerability assessment (33%) as part of university projects. Few students (11%) performed vulnerability detection outside of university projects, or (7%) identified them for several projects.

We investigated how many vulnerabilities (both for the cases $\delta = 0$ and $\delta = 3$) each participant found. For $\delta = 0$, 29 students (11% of the total number of students) did not detect any vulnerability, which corresponds to 58 data participants, vulnerability pair, instead, for $\delta = 3$ only 11 students ((4%) of the total number of students) identified zero vulnerabilities corresponding to 44 data participant, vulnerability pair. Therefore, we consider our population a good sample to answer our research questions because the vast majority of people find some vulnerabilities in the code.

### RQ1. Choosing the right $\delta$

In this section, we denote the number of pairs *(participant, vulnerability scenario)* for which zero vulnerable lines were found by $\#(TP = 0)$, and for which some vulnerable lines were found by $\#(TP > 0)$

**Fig. 5:**
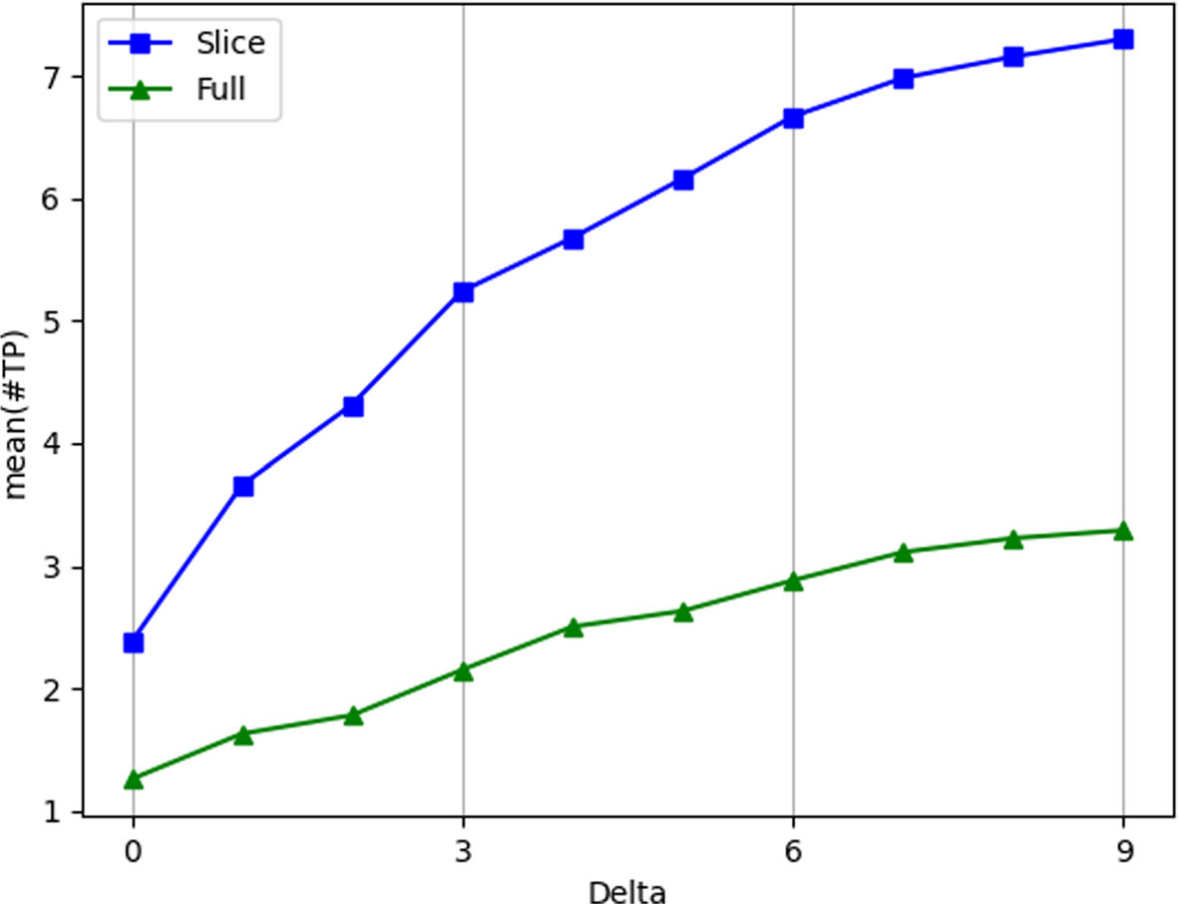
TP across all vulnerabilities grouped by file type. The figure shows on the y axis the mean of the number of TP lines found across all the pairs *(participant, vulnerability scenario)*

Figure 5 shows how the TP mean across all vulnerabilities varies for different values of ${\delta }$-neighborhood from 1 to 9. We observe that values of $\delta > 3$ lead to a too large number of TPs, resulting in overoptimistic results. To answer RQ2(§ 5.3) and RQ3(§ 5.4) we decided to analyze the data for $\delta = 0$ because it represents the case in which the participants identify exactly the vulnerable lines, and for $\delta = 3$ as it corresponds to the case in which the participants identified the vulnerable area around the ground truth vulnerable lines. Moreover, as we mentioned in Section 1$\delta = 3$ is the same value used for the command git diff^4^.

### RQ2. Slicing effectiveness

Table 5 shows the number of (participant, vulnerability scenario) pairs for which zero vulnerable lines were found, (denoted by $\#(TP = 0)$ and for which some vulnerable lines were found (denoted by $\#(TP > 0)$ for $\delta = 0$ and $\delta = 3$ for the original files treatment, and the sliced files treatment. In the following section, we first report the results obtained for $\delta = 0$, and then for $\delta = 3$.

**Table 5:** Number of $\#(TP_{\delta }=0)$ and $\#(TP_{\delta }>0)$ per vulnerability

	Original		Slice	
$$\delta = 0$$				
	$\#(TP = 0)$	$\#(TP > 0)$	$\#(TP = 0)$	$\#(TP > 0)$
path	85	47	36	96
user	70	63	29	102
XSS	89	44	61	70
DoS	105	25	83	51
Total	349	179	209	319
$$\delta = 3$$				
	$\#(TP = 0)$	$\#(TP > 0)$	$\#(TP = 0)$	$\#(TP > 0)$
path	82	50	12	120
user	61	72	12	119
XSS	62	71	44	87
DoS	102	28	81	53
Total	307	221	149	379

For each vulnerability (the table’s rows), we report in the second and third columns the numbers $TP_{\delta =[0,3]}=0$ and $TP_{\delta =[0,3]}>0$ for the original file. Instead, the third and fourth columns report the number $TP_{\delta =[0,3]}=0$ and $TP_{\delta =[0,3]}>0$ for the sliced file

#### Identifying the exact vulnerable lines ($\mathbf {\delta } = \textbf{0}$)

. We first used a Chi-squared test on the overall findings (values from the total row from Table 5). We obtained $\chi ^2_{\delta =0}(1056, df=1)= 73.42, p = 1.05 \cdot 10^{-17}$ with an odds ratio of 2.98x, so it is three times easier to find a vulnerability when inspecting a slice of the original code.

Using the Agresti-Coull-Wilson rule for confidence interval (Agresti and Coull 1998), the 95% C.I. of the probability of identifying the vulnerability if the original file is given to a participant is [0.29, 0.38] while the 95% C.I. of the probability of identifying it if a sliced file is supplied is [0.56, 0.65].

We also performed two Chi-squared tests on the two different groups (original treatment vs slice treatment) to verify if there is a difference between the types of vulnerabilities. For the original files we obtained $\chi ^2_{\delta =0}(528, df=3) = 23.46, p = 3.24 \cdot 10^{-05}$. The odds ratios are not very different (all just above two): using the findings on the DoS vulnerability as a reference we found that only for user vulnerability the odds ratio is larger than three (3.8x).

For the sliced files we obtained $\chi ^2(528, df=3) = 55.71, p= 4.84 \cdot 10^{-12}$. The odds ratios are slightly different: using again the findings on DoS as a reference line we found that finding the user vulnerability is easier with an odds ratio of 5.7x.

We further investigated these results by analyzing only the data *(participant, vulnerability scenario)* pairs with $\#(TP > 0)$. We performed a test of normality on each vector for the two different treatments (i.e. one array for the original files, and one array for the slice files), Removing the data *(participant, vulnerability scenario)* pairs for which TP is equal zero. We obtained $\mathcal {N}(179) = 45.42, p = 1.37 \cdot 10^{-10}$ for the original files, and $\mathcal {N}(319) = 81.83, p = 1.70 \cdot 10^{-18}$ for the slice files. Finally, we used TOST to investigate the similarity of the two groups. We used the Mann-Whitney U test ($lowerbound = 0.8$) to check the equivalence between the means of the two samples. As result we obtained $U_{lower\_bound} = 20931, p = 2.34 \cdot 10^{-07}, U_{upper\_bound} = 17397, p = 8.09 \cdot 10^{-14}$. So the sliced and original files are statistically equivalent.

From the results we obtained for $\delta = 0$, we can conclude that slicing is only useful for ‘finding something’ as opposed to ‘finding nothing’. When we say *“once participants find something there is no difference between inspecting the original file or a slice”*, we consider the code inspection scenario in which a code reviewer has identified *some* of the vulnerable lines ($\#(TP_{\delta } > 0)$). Of course there might be cases in which the participants have identified just one line $TP_{\delta } =1$ or cases in which the participants have identified all vulnerable lines $TP_{\delta } =GT$. A priori, before running the experiment, slicing could have helped also in this case and participants exposed to the slices could have found significantly more vulnerable lines than participants exposed to the full file. Once we consider only the participants that identified at least one vulnerable line (i.e. we exclude the participant data point with $\#(TP_\delta =0)$ for a specific vulnerability), there is a significant equivalence between the two groups of participants with $\#(TP_{\delta } > 0)$ for the slice, and of participants with $\#(TP_{\delta } > 0)$ for the full. Therefore, once a participant ‘found something’ (at least one vulnerable line of that type), the number of additionally found lines (TPs) on a slice are statistically equivalent to the number of additionally found lines (TPs) on a full file. This scenario can be compared to the first step of fault localization which is identifying whether there is a vulnerability. The second scenario which is identifying all lines characterizing the vulnerability is the second phase of fault localization.

#### Identifying the vulnerable area ($\mathbf {\delta } = \textbf{3}$

. As for $\delta = 0$, we first performed a Chi-squared test on the overall findings and we obtained $\chi ^2_{\delta =3}(1056, df=1)= 95.14, p = 1.77 \cdot 10^{-22}$ with an odds ration of 3.5x, so it is three and a half time easier to find a vulnerability when inspecting a slice of the original code.

When using the Agresti-Coull-Wilson rule, the 95% C.I. of the probability of identifying the vulnerability if the original file is given to a participant is [0.38, 0.46] while the 95% C.I. probability of identifying it if a sliced file is supplied is [0.68, 0.75].

The Chi-squared test’s results for the original files is $\chi ^2_{\delta =0}(528, df=3) = 38.41, p = 2.31 \cdot 10^{-08}$. The odds ratios (using DoS vulnerability as baseline), are not different for the vulnerabilities user and XSS (just above four). Instead, we found that for the vulnerability path, there is an odds ratio of 2.2x.

For the sliced files we obtained $\chi ^2(528, df=3) = 117.91, p= 2.18 \cdot 10^{-25}$. Using again DoS as a reference line, the odds ratios for path and user are just above fifteen, instead for the vulnerability XSS the odds ratio is 3x.

We then analyzed only the data for $\#(TP > 0)$, and we performed a test of normality for the two different treatments (original vs. slice). We obtained $\mathcal {N}(221) = 48.45, p = 3.02 \cdot 10^{-11}$ for the original files, and $\mathcal {N}(379) = 133.15, p = 1.22 \cdot 10^{-29}$ for the slice files. Finally, we used TOST to investigate the similarity of the two groups. We used the Mann-Whitney U test ($lowerbound = 0.8$) to check the equivalence between the means of the two samples. As result we obtained $U_{lower\_bound} = 50080, p = 0.99, U_{upper\_bound} = 17364, p = 6.77 \cdot 10^{-34}$. Contrarily from the result we obtained with $\delta = 0$, the two groups are not statistically equivalent.

With $\delta = 3$, as for $\delta = 0$, we observe that slicing is useful to ‘find something’ as the Chi-squared test is statistically significant. However, contrarily from the observations for $\delta = 0$, when we consider the participants that found at least one vulnerability, for slicing treatments more vulnerabilities have been identified. This result aligns with our expectations as inspecting fewer lines of code should make it easier to identify a vulnerability.

We further discuss these results in Section 8.

### RQ3. Slicing usefulness for each vulnerability type

Table 6 shows the mean value and the standard deviation (in the brackets) for the number of TP, FP, and FN that respect the condition $TP > 0$. Instead, Table 7 shows the result of the four TOST tests that we performed for each vulnerability type. We report and discuss these results first for $\delta = 0$, and then for $\delta = 3$.

**Table 6:** Table descriptive statistics of TP greater than zero

	*n*	$TP\mu (\sigma )$	$FP\mu (\sigma )$	$FN\mu (\sigma )$
$$\delta = 0$$				
Original				
path	47	2.17 (1.46)	3.18 (2.79)	12.23 (1.36)
user	63	1.56 (0.87)	2.47 (2.14)	4.29 (0.91)
XSS	44	2.02 (0.84)	3.09 (3.61)	3.36 (1.03)
DoS	25	1.72 (0.66)	3.00 (2.69)	2.73 (0.62)
TOT	179			
Slice				
path	96	2.11 (1.38)	2.02 (1.21)	11.46 (1.51)
user	102	1.93 (0.98)	1.71 (1.00)	3.55 (1.11)
XSS	70	1.99 (1.13)	2.25 (1.54)	3.24 (0.93)
DoS	51	1.75 (0.65)	2.46 (1.72)	2.44 (0.81)
TOT	319			
$$\delta = 3$$				
Original				
path	50	3.20 (1.89)	2.28 (3.42)	0.003 (0.05)
user	72	2.00 (0.99)	1.38 (1.97)	0.003 (0.05)
XSS	71	2.66 (1.05)	1.92 (4.03)	0.002 (0.04)
DoS	28	2.68 (0.54)	1.71 (2.25)	0.001 (0.03)
TOT	221			
Slice				
path	120	4.61 (2.55)	0.28 (0.57)	0.005 (0.07)
user	119	3.14 (1.29)	0.41 (0.59)	0.003 (0.05)
XSS	87	3.44 (1.16)	1.49 (1.56)	0.002 (0.04)
DoS	53	2.96 (0.19)	1.42 (1.59)	0.0002 (0.01)
TOT	379			

For each vulnerability (rows of the table) we report the mean value of TP, FP, and FN and the standard deviation value (in the brackets) for the original files. We report the same metrics for the sliced files. At the top of the table, we report the results for $\delta = 0$, and at the bottom for $\delta = 3$, and we consider only the *n* data point with $\#(TP_{\delta =3} > 0)$, which are the second and fourth column of Table 5. While the original data was balanced, the number of files in which something was found by slicing is higher than the number of original files in which at least one vulnerability was found, as implied by RQ2

**Table 7:** Equivalent test results for each vulnerability type

	Lower Bound		Upper Bound		
	statistic	p-value	statistic	p-value	equivalent
$$\delta = 0$$					
path	1597	1.95e-03	1553	1.04e-03	yes
user	2821	0.08	1294	2.45e-11	no
XSS	994	6.14e-04	1236	0.03	yes
DoS	392	2.58e-03	363	8.79e-04	yes
$$\delta = 3$$					
path	3429	0.92	1436	3.84e-08	no
user	5555	0.99	1193	1.29e-17	no
XSS	3467	0.91	1014	1.19e-14	no
DoS	410	6.14e-05	40	2.21e-16	yes

For each vulnerability type, we performed a TOST test for equivalence comparing the original files with the sliced files by considering the number of $TP_{\delta =3} >0$. For each vulnerability, we report the results of the Mann-Whitney U test for both lower bound and upper bound. We can observe that there is essentially no difference between the two sample groups for the vulnerabilities path, user, XSS. Instead, there is a difference for the vulnerability DoS which is only equivalent at the 10% confidence level for the lower bound

#### Identifying the exact vulnerable lines ($\mathbf {\delta } = \textbf{0}$)

Table 6 shows the mean and standard deviation of TPs, FPs, and FNs, for the two treatments (original files vs sliced files) considering $TP_{\delta = 0} > 0$. We notice that the TP’s mean values from original and slice are close, and there is no increment in the values. This result is in line with the findings of RQ2 for $\delta = 0$. We further investigated the data by using TOST to verify whether slicing intervention is more effective in identifying certain types of vulnerabilities than others. As in Section 5.3 we used the Mann-Whithney U test ($lowerbound = 0.8$ ) to check the similarity between the samples. We report the results of the four TOST tests for each vulnerability type for $\delta = 0$ in Table 7. We observe that for the vulnerabilities path, XSS, and DoS there is essentially no difference, except for the user vulnerability. This could be explained by the fact that the user vulnerability is characterized by a specific pattern, therefore, it is easier for the participants to identify it when inspecting fewer lines of code.

#### Identifying the vulnerable area ($\mathbf {\delta } = \textbf{3}$)

. Contrarily from the findings with $\delta = 0$, in Table 6 we can notice an increment of the TP’s mean values for the slice treatment, therefore, it suggests slicing intervention is effective in identifying certain types of vulnerabilities. Again we used TOST for further investigation, and from the results reported in Table 7 we can notice there is a difference for the vulnerabilities path, user, XSS. Instead, as for $\delta = 0$, there is no difference between the two groups for the DoS vulnerability. Despite this case which could be explained by the fact that normally a DoS vulnerability is not characterized by a specific pattern, therefore it is harder to identify it, the findings are in line with those from RQ2.

## Qualitative analysis of Participants Justifications

We collected the participants’ motivations for the last two years we performed the experiment and in this section we report the result of a coding analysis of these justifications.

### Codebook Development

To analyze the motivation responses of the participants, we have adopted the *applied thematic analysis* (Guest 2012), following the principle of emergence (Gregory et al. 2015), according to which the data gain their relevance in the analysis through systematic generation and iterative conceptualization of codes and concepts. Eventually, all justifications are analyzed and classified according to the categories (codes) so identified.

During a first phase, the first and third author reviewed jointly a samples of justifications for each vulnerable scenario to identify a first set of emerging codes. Those were consolidated into a codding book which is reported in Table 8. It lists the codes identified in our study, also showing some examples.

**Table 8:** Codes used in the study

Code	Guide	Example
Methods,Variables	Identifiers of the fragment of the program are mentioned (e.g. a specific variable name, a method name etc.)	"uri", thus "mapuri", thus "child" path is not normalized. This could be exploited with a path traversal attack.
Lines number	A specific line number is mentioned	normalize checks if "/../" appears at the beginning of the string but it doesn’t check if "/../" appears in other parts of the string,which could lead to a path traversal. Line 76 normalize is called and line 95 returns object based on unsafe path
Relevant keywords	Some keyword related to the type of attack are mentioned.	I think that these lines of code are vulnerable because the code takes as input a path and replies with an URL to that resource. It could be possible for an user to input a specific path that will be processed by the code incorrectly.
Vulnerability text	The text is specific to the vulnerability in question	Line 144 - Injection: the function authenticate accepts user input without sanitization and uses it to access the data storage. Lines 186-191 and 195 - Injection: the function addUser doesn’t sanitize the inputs and adds them to the storage
Security text	The text is security related but it is not the right answer	65: Validated is not explicitly initialized as false before the check is performed, 62: if plaintext string ends up being compared, can’t ignore case; 93: wild guess that the md refers to MD hashing algorithm which isn’t secure
Potential exploit	The possible attack that could be carried out is mentioned	Line 144 - Injection: the function authenticate accepts user input without sanitization and uses it to access the data storage. Lines 186-191 and 195 - Injection: the function addUser doesn’t sanitize the inputs and adds them to the storage
I don’t find it	The participant does not find the vulnerability	Couldn’t find any vulnerable lines
Unclear answer	The response is too vague and generic.	Information Disclosure

Then the second author confirmed the coding book guidelines by further checking a smaller additional sample. Finally, the first author marked all justifications and an independent researcher, not author of this paper, checked the coded justifications. Additional conflicts where discussed by the first and third author. All coded justifications were eventually agreed. To avoid any confirmation bias the coders only looked at the information on the type of vulnerability (e.g. XSS vs Path Traversal) *without knowing* which lines were formally inserted in the response, and whether the lines proposed by the participant were actually vulnerable or not according to the ground truth.

Several of the codes are self evident: *Methods/Variables identifiers are mentioned* or *Lines number are mentioned* mark justifications in which specific identifiers from the source codes or specific lines are mentioned in the justifications. It is possible that the lines mentioned by the participants in the justifications would be different from the lines they inserted in the formal response. This is immaterial to the idea behind the code that they have considered specific fragment of code in their reasoning (wrong or right as they might have been).

The idea behind the code *Relevant Keywords are mentioned* has also been used in industry to identify vulnerability fixing commits when mining software repositories (Sabetta 2024). The key idea is that the justification mentions some specific keywords related to the type of attack that are not necessarily identifier mentioned in the source code. For user injection, we expect the presence of keywords about ’user controlled input’ and ’sanitization’. For XSS, we expect some text mentioning ’HTTP’ or ’forms’ being manipulated. For path traversal, we should have some sentences mentioning paths and accessing directories. For DoS something about buffer overflow or not controlling loop variables or anything related to disrupt a service.

**Table 9:** Co-occurences of codes across responses

	% Total	V.M.	L.N.	R.K.	T.V.	T.S.	P.E.	N.F.	U.A.
V.M.	34%	100%	41%	**78%**	**69%**	27%	12%	0%	2%
L.N.	39%	37%	100%	**73%**	**63%**	31%	10%	0%	4%
R.K.	72%	38%	40%	100%	**77%**	20%	10%	0%	1%
T.V	56%	42%	44%	**98%**	100%	0%	13%	0%	0%
T.S.	32%	29%	37%	45%	0%	100%	3%	0%	0%
P.E.	8%	49%	46%	**91%**	**86%**	11%	100%	0%	0%
N.F.	1.2%	0%	0%	0%	0%	0%	0%	100%	0%
U.A.	9%	8%	19%	8%	0%	0%	0%	0%	100%

The table shows the co-occurences of codes among the population, each row and column (2-9) represent a rule: variable and methods (V.M.), line numbers (L.N.), relevant keywords (R.K.), text related to the vulnerability (T.V.), text related to security (T.S.), potential exploit (P.E.), I don’t find it (N.F.), and unclear answer (U.A.). The second column shows the percentage of total responses that check a specific rule. Each cell of the table is the percentage of responses for a specific co-occurence rules. For example, 49% of the responses that mention a possible exploit (P.E. row), also mention variables or methods identifiers (V.M. column)

The difference between the *Text is related to the specific vulnerability* and *Text related to security* is subtle but important. The former code marks justifications where the response is related to the vulnerability in question (e.g. the text is unmistakably about XSS and the vulnerability is about XSS), and it is clear that the participant has knowledge of the task is performing and the vulnerability is looking for. The participants might have still identified the wrong lines according to our ground truth but still had some very precise idea of what was doing. The latter code captures cases in which the participant has the clearly made a mistake as the explanation is not related to the specific vulnerability, but they are still mentioning a security issue, that is *a plausiblevulnerability* given the code fragment that they had, and considering that they could not actually run tests. For example a participant analyzing a DoS might spotted the absence of a finally block in try-catch Java fragment, even though that particular fragment did not correspond to a vulnerability. It shows that even though the participant got the wrong response, they answered at their best capabilities and in a sensible way. The *Potential exploit is mentioned* is used when the justification includes additional information on the malicious action that could lead to a possible attack specific for the vulnerability.

The remaining two codes *I don’t find it* and *Unclear answer* are used to distinguish the cases in which no code applies because the participant didn’t find it from the case in which no code applies because the response is a gibberish explanation.

### Findings

Table 9 shows the co-occurences of codes among our population. We can observe that 78% of the participants mentioning variables and methods names, they also mention keywords. For example one participant when motivating the answer for the XSS vulnerability, wrote: *The servletRelativeAction parameter is retrieved from user input (i.e., it’s a request parameter) and is used to build a URL. For instance, if the user submits a malicious input like " &gt; &lt;script&gt;alert(’XSS’) &lt;/script&gt;, it can cause a XSS vulnerability.* When referring to variables and methods, 41% also mention line numbers, for example one participant wrote: *109 - the variable pos in the while loop is obtained from the header which can be modified by the user and exploited; 187 - again an array is created using the pos variable; 228 - the same variable in 187 is used to modify another array here*, referring to the path traversal vulnerability.

In Table 10 we show the number of responses categorized by the number of words used, and “quality code” checked (i.e. all codes excluding *I don’t find it* and *unclear answer*). We can observe that a total 78% of explantions were 10 words or longer, and over 30% of the explanations were longer than 30 words. Over 80% of explanations had two or more quality codes (e.g. mentioned vulnerable lines and related security keywords) and more than 40% of the explanations had more than 3 quality codes.

**Table 10:** Quality indicators across responses

#Word	#Responses		#Quality Code	#Responses	
<10	100	22%	No code	35	8%
10-19	121	26%	1 code	49	11%
20-29	95	21%	2 codes	159	35%
30-39	94	21%	3 codes	145	32%
40+	46	10%	4+ codes	68	14%
Total	456		Total	456	

The first column lists a group of number of words intervals, and the second column is the total number of responses for each category #Words. The third column is the percentage among the total. The fourth column describes the category of “quality codes” found in a response. The quality code categories do not include the codes *I don’t find it* and *Unclear answer*. In the fifth column we report the total of responses for each code quality, and in the last column the percentage among the total

## Perception analysis

We performed a perception analysis to assess the quality of the results and to ensure that the experiment did not suffer from internal validity issues. In particular, we asked whether the participants (1) had a clear understanding of the experiment tasks, (2) had enough time to complete the experiment, and (3) the training was sufficient to carry out the tasks.

To collect the variables *Knowledge of Java* and *Knowledge of Security Vulnerabilities* we used a scale from 1 to 4. The lowest value is “participant does not have any experience", followed by “participant has some knowledge from a university course", then “participant did few projects (or an internship)", up to the highest value which is “participant did several projects outside of university context".

To collect perception information about the time, and the training material provided we used Likert scale from 1 (strongly disagree) to 5 (strongly agree) (Joshi et al. 2015). We have not relied on comparing participant background knowledge with the perception questions, instead we have investigated the impact of background knowledge on the results.

Finally, we gave the participants the opportunity, through an open box question, to leave any comment or feedback they had in general about the experiment.

We also collected general opinions on the experiment. Most of the participants agreed (61%) and strongly agreed (21%) that they understood the task they had to perform for the experiment. A few percent strongly disagreed (2%) and disagreed (4%) that the explanation of the experiment was clear. Few participants (12%) neither agreed nor disagreed. For example, a participant correctly detected the vulnerable lines for the CVE-2012-2733 providing a proper justification: *“The loop is controlled by the length of the headers, can lead to a DoS”*, which is in line with the answer given by another participant: *“The loop for reading the header depends on the how many headers are there in user’s data. A huge amount of headers can bring down the server”*. Another participant reported the right justification after correctly detecting the vulnerable lines for the CVE-2008-2370 on the slice file: *“normalize checks if "/../" appears at the beginning of the string but it doesn’t check if "/../" appears in other parts of the string, which could lead to a path traversal. Line 76 normalize is called and line 95 returns an object based on an unsafe path”*

The participants agreed (52%) and strongly agreed (24%) that they had enough time to perform the experiment. Only 1% strongly disagreed, and 4% disagreed that the time was not enough. Finally, 19% of the participants neither agreed nor disagreed.

Some participants suggested that more detailed training could be beneficial, and more examples could be provided. While 28% either agreed or strongly agreed that the training was sufficient, 36% of the participants neither agreed nor disagreed. One of the participants declared that *“I believe that more examples should have been given, since in the explanations we only got very low-level examples. They were not enough to get a good enough understanding of the type of vulnerabilities we would get.”*; Another one suggested *“Java docs could have been useful (for example trim, lastIndexOf...) just to be sure about the code behavior.”*, which is inline with another participants opinion: *“Running the code would be helpful. Otherwise access to java documentation.”*.

## Discussions and implications

In real-world scenarios, code inspectors often approach a codebase without the certainty that vulnerabilities are present, and the challenge lies in both detecting and precisely locating any potential weaknesses, and actually understanding what the problem is. The study’s approach, where the existence of a vulnerability is a given, could have influenced participants’ behavior and perceptions, potentially making them more alert and focused solely on pinpointing its location. However, with our experiment we have been able to separate the problems in two parts, and focus only on the challenge of understanding where weakness locally is. We wanted to understand whether simplifying the code by removing the irrelevant lines would help code reviewers to identify vulnerabilities (assuming to have the perfect line identification algorithm).

Another fundamental point that we wanted to investigate was also whether the influence of the participants’ background (e.g. knowledge of Java, knowledge of vulnerability detection) have a significant impact on the results that we obtained. We performed a Chi-squared test to check the correlation between the number of TPs and both the Java Knowledge and Vulnerability Detection. However, none of them resulted to be significant.

Despite the results obtained with the Chi-squared test, we decided to exclude the participants without knowledge of Java, as we thought that it is a minimum requirement to understand the code and perform the tasks. Therefore, as we already mentioned in Section 5, we analyzed the data and reported the results excluding the participants with no experience in Java. However, we also performed a general analysis including the students without experience in Java. The results do not statistically differ from the ones reported in Section 5.3 and Table 6. There is a small difference with the results from the ones of Table 7. When including people with no experience in Java, the two sample groups do not result equivalent for the vulnerability path. However, it is a small difference as the value for the lower bond is $MWU = 2531, p = 0.076$ and $MWU = 2081, p = 0.0013$ for the upper bound.

The findings in Section 5.2 suggest that summary indicators (such as Precision, Recall and Jaccard index) that are reliable when the numerator and denominator are large numbers derived from large samples, may be unreliable when dealing with smaller samples, which is commonly the case for vulnerability code inspections performed by human assessors. In such context, counting lines that identify a *region* as TPs may be more representative. In future experiments, researchers may opt for a base decision rule.

From the results that we reported in Section 5.3 we observed different outcomes for $\delta = 0$ and $\delta = 3$. In both cases, the Chi-squared test is statistical significant when comparing the overall TPs for the two sample groups (original vs slice). The results suggest that more vulnerabilities have been identified when inspecting the sliced files. As there is a large number of $TP = 0$, we decided to make a comparison of the data, considering only the data points with $TP > 0$, and in this case, the findings between $\delta = 0$ and $\delta = 3$ differ. In the first case ($\delta = 0$) which correspond to the scenario of matching the exact ground truth vulnerable lines, the TOST test results significant, therefore, the two sample groups (original vs slice) are equivalent. This result shows that it is easier to identify a vulnerability when inspecting a sliced file, however, when we analyze only the data points for which at least one vulnerability has been detected, slicing does not help to identify more vulnerabilities. A possible explanation is that for a human assessor matching the exact lines of the ground truth is harder. Instead, when we consider the scenario of identifying the vulnerable area ($\delta = 3$), the two sample groups do not result equivalent. This indicates that more vulnerabilities are detected when inspecting the sliced files, compared to the full files. This finding is expected, as it should be easier for a human assessor to identify the vulnerability when inspecting fewer lines of code.

In Section 5.4 we analyzed the data to determine if there is any difference in the number of TPs among the different type of vulnerabilities between the two different treatments (original vs slice). We performed four TOST tests to compare the two different treatments (one for each vulnerability type), and for $\delta = 0$ we observed that the two sample groups differ only for the user vulnerability. Instead, for the other vulnerabilities there is an equivalence. We think this result can be explained by the fact that the user vulnerability is easier to be identified, especially when inspecting few lines of code, as it characterized by a specific pattern. We performed the same statistical tests with $\delta = 0$ and in this case we observed an equivalence between the two sample groups only for the DoS vulnerability. This is an expected result it seems obvious that is easier to identify the vulnerable area when inspecting less line of code, expect for the DoS vulnerability which it might be harder to be identified from the participants.

## Threats to validity

### Population

#### Threat

We acknowledge that the background and practices of the participants may impact the experiment’s results. Still, several studies with promising results involving students have been performed  (Chong et al. 2021; Naiakshina et al. 2017, 2018; Rong et al. 2012). Indeed, the study performed by Salman et al. (2015) compared the performances between students and professional to investigate how well students represent professionals as experimental subjects in SE research. When approaching something new for the first time, the performance is similar for both subject groups so results might not be transferable to people with significant code inspection expertise (but they might not need slicing anyhow). Tahaei and Vaniea (2022) performed an experiment to explore the programming skills, privacy and security attitudes, and secure development self-efficacy of participants from a CS student mailing list and four crowdsourcing platforms (Appen, Clickworker, MTurk, and Prolific). The results show that 89% of CS students answered all programming skill questions correctly compared to 27% of crowdsourcing participants.

#### Mitigation

Due to our study population, the findings of this paper are limited to the educational context, and may not be representative of all real-world scenarios. Replicating the same study with experienced professionals could lead to different results due to their background, or experience. Therefore, more studies should be performed to get closer to reality.

### Contamination by results from previous year

#### Threat

Through the years that we performed the experiments we used the same vulnerabilities dataset. This choice might arise a contamination effect on participants of year N by the results from year N-1.

#### Mitigation

As the successful execution of the experiment does not affect the grade of the students to pass the course, the students are not incentivized in sharing the results between them. Moreover when the students were asked to analyze the data of their fellows, we made available their data, but also the data from the previous years. Also, we did not observe a significant improvement of the results through the years, therefore, we think that the participants are not affected by the results from the previous years.

### Usage of checklists

#### Threat

Checklists may lead to a fixation effect (Sio et al. 2015), inducing the participant to focus on specific patterns to identify vulnerabilities, with the risk of having negative results.

#### Mitigation

We have provided general guidelines and made sure these were clear to the participants to avoid such effects.

### Realism

#### Threat

The study’s approach, where the existence of a vulnerability is a given, could have influenced participants’ behavior and perceptions, potentially making them more alert and focused solely on pinpointing its location.

#### Mitigation

As our goal was to investigate the effectiveness of method-level slicing in code review to identify vulnerabilities, we think that this threat does not influence the results obtained. Moreover, the student did not have prior knowledge that two files were a slice of code. They performed the experiment with the only purpose of identifying the vulnerabilities. This design has been chosen also to provide a fair evaluation in a class setting.

### Generalizability of the results

#### Threat

The restricted selection of the vulnerabilities may affect the generalizability of the results as we selected four specific types. In addition, we consider the risk of our results generalizing beyond the adopted slicing algorithm and code project.

#### Mitigation

To reduce the impact of this threat we selected the most common type of vulnerabilities at the time of study conceptualization. In the future more experiments with different vulnerabilities, slicing algorithms, and code projects can be performed to further analyze and improve the generalizability of the results. Moreover, we could try to add more example with multiple vulnerable location in the files to very if there is a difference when identifying the vulnerabilities.

### Choice of slicing algorithm and defining the ground truth

#### Threat

In our design we provide the ‘perfect’ seed lines to the slicing algorithm, therefore, the participants do not have to create the sliced code, even though in a real world scenarios developers compute the slices by themselves by identifying the suspicious zones. We use thin slicing (Sridharan et al. 2007) as implemented by Dashevskyi et al. (2018) to extend the SZZ algorithm for vulnerability analysis (Bao et al. 2022). However, one of the main challenges we faced was identifying the seed to produce the sliced files from the algorithm by Dashevskyi et al. (2018) used as a screening test for vulnerable commits. Hence, the identification of the ‘perfect’ seed might be a confounding factor.

#### Mitigation

Two security experts collaborated together to define the ground truth and define the seed. In addition, the security experts made sure the slicing algorithm preserved the vulnerable lines identified when producing the slices. A similar approach is used in Bui et al. (2024) to identify the vulnerable lines that has to be fixed. The approach used in Dashevskyi et al. (2018), given a known vulnerability that is already fixed, helps developers to know whether their code is affected and what they need to change accordingly. We chose this algorithm because it is lightweight and does not produce executable slices of code, which was not necessary to perform the experiment. Other tools like JoanAudit (Thomé et al. 2017) simply creates an HTML report to guide security auditors audit the source code.

## Conclusions and future work

In this paper, we presented a controlled experiment to investigate whether slicing intervention supports developers in detecting security vulnerabilities during code inspection.

Our population was composed of 264 master’s computer science students who had to identify four different types of vulnerabilities that we selected among the OWASP Top 10 (path, user, XSS, DoS). With a balanced design, six different treatments that differed in the pair *(Type of Vulnerability, Original/Sliced code)* were presented to the participants. To create the sliced files we used the algorithm by Dashevskyi et al. (2018) used as a screening test for vulnerable commits. We also designed a methodology to define whether considering a participant’s response correct by exploring different notions of neighborhood to define the correctly identified lines.

By using a notion of $\delta$ neighborhood we analyzed the data for $\delta = 0$ which corresponds to the case of exactly identifying the vulnerable lines, and for $\delta = 3$ which represents the case of identifying the vulnerable area on the context size of the command git  diff. In both cases we found that slicing helps in in ‘finding something’ as opposed to ‘finding nothing’. However, for the case of $\delta = 0$, we also found that once ‘some’ correct lines have been identified, slicing makes no significant difference in the number of correctly identified fragments, expect for the user vulnerability. Instead, for the case of $\delta = 3$ there is a difference as we would expect since analyzing less line of code should make easier to identify the vulnerability.

More experiments with more vulnerability types and files are needed to determine whether this result is due to the different types or just our choices (e.g. our choice of using Java as the language of choice). We acknowledge this study is performed in a research context, and the study’s population is master’s student, however, we believe these results are promising, and open new exploring directions to further analyze the implications reported in this paper.

Such experiment could be also replicated in other contexts as within companies with professional developers to investigate slicing intervention in another context rather than research, and make this study also applicable to the reality and generalize the results obtained. For example, practitioners could use git diff, but, since secure development practices vary in organizations, determining a good decision rule might require considering the security code review process at the company, and sound-boarding the rule with several domain experts, as suggested by Braz et al. (2022). In addition, since code reviewers tend to find more vulnerabilities compared to developers (in a study of Yu et al. 2023, only 12% were found by developers and 88% by reviewers), in scenarios where developers are "finding nothing", slicing might help. Finally, some vulnerabilities might be easier to identify as they are characterized by a pattern, therefore in this case practitioners should not encounter too many difficulties when performing code review. For the other vulnerabilities that are harder to identify, companies should invest in slicing tools and training to support code reviewers and developers.

## Footnotes

1 https://owasp.org/www-project-top-ten/2017/Top_10

2 https://www.secura.com/services/people/training-courses/secure-programming-training

3 https://owasp.org/www-project-top-ten/2017/Top_10

4 https://git-scm.com/docs/git-diff
